# The Royal Hospital for Incurables, Putney

**Published:** 1892-08-06

**Authors:** 


### THE ROYAL HOSPITAL FOR INCURABLES, PUTNEY.

Last week we published the findings of the Lords Committee so far as this hospital is concerned. The following is the "answer of the Board of Management to the recommendations contained in the report of the Select Committee of the House of Lords, 1892." The "answer" is dated July 22nd, and is signed on behalf of the Board by Mr. John D. Allcroft, Treasurer :—

When the Select Committee of the House of Lords opened their inquiry into the conduct of the Royal Hospital for Incurables at Putney, two facts became clear.

1st. That their Lordships evidently did not understand the nature of the institution; they had not been to see it, and the notion of a General Hospital or a Poor Law Infirmary was the test by which it was judged.

2nd. That the inquiry was not to be exhaustive.

It was also evident that their Lordships had been supplied with information, of what exact nature, or by whom furnished, did not transpire, dealing with circumstances and cases covering many years. The information thus obtained was put into the form of leading questions, to which the Secretary, who was the only witness examined, was expected to reply in the affirmative or negative, without being allowed to give any explanations. The management was thus placed on its trial, and the witness had to answer numerous questions without preparation and from memory.

The Board, whilst receiving with all respect the suggestions of the Lords' Committee, feel that it is scarcely reasonable that they should accept theories of domestic management from those who have not set foot within the establishment they assume to reform. It must be borne in mind that the Board, who have devoted years of labour and thought to this Home, claim that their experiences are at least entitled to as great consideration as the theories of the Select Committee.

They are now called upon to reply to the criticisms of that Committee; they would have preferred doing so at the annual meeting of Governors, but that will not take place till the end of November. In the meantime, they submit to their subscribers and the public the remarks that follow.

For convenience, the report of the Lords' Committee will be considered under its several recommendations.

I.—*That a resident Medical Officer should be appointed with general control in the absence of the Committee, as is the case in the Poor Law Infirmaries.*

Though the Board do not concur with their Lordships as to a resident medical officer, they feel that the question is one of the gravest possible import, and are taking steps to obtain the best medical opinions they can on the subject.

Upon the question itself, the Board observe that the standard or ideal proposed by the Lords' Committee in this recommendation, is that of the Poor Law Infirmary. The want of analogy is easily discovered by any person acquainted with the object of the Royal Hospital for Incurables, and the way in which it has been sought to realise that object. It is not allied either to a general hospital on the one hand, or to a Poor Law Infirmary on the other; being, in fact, a Home for Chronic Invalids. Of these a large proportion are suffering from rheumatism or paralysis, not requiring active medical treatment, their principal need being quiet and careful attendance. The average number calling for specific medical treatment is about seven persons per day, or less than four per cent. of the whole.* It is difficult therefore to see that the continual presence and the devotion of the whole time and energy of a professional man are needed.

By the existing arrangement a medical man, living in the immediate neighbourhood, visits the institution every week day, and sees such of the inmates as require his services. In the event of emergencies, which, from the chronic and passive nature of the maladies, are infrequent, the Matron (who is a Nightingale Nurse) and a staff of hospital-trained nurses are always on the spot to attend to them. In fact, as shown in the evidence given before the Lords' Committee, the inmates are as well cared for in a medical point of view as members of a private family who are able to pay substantial fees.

It is to be noted that no evidence was adduced before that Committee, who it appeared had been supplied with information in great variety, counter to the above statement of facts. At the same time, the only witness who was summoned, explained fully the difference between this home and a general hospital, where accidents and acute diseases are received; and he moreover invited their Lordships to pay it a visit, which was unfortunately declined.

II.—*That a Ladies' Committee should be appointed as a large majority of the patients are females.*

The Board are of opinion that such a dual administration would be likely to result in difficulties and confusion; and therefore cannot see their way to adopt their Lordships' suggestion.

They are thankful to know that a great number of ladies are in the habit of regularly visiting the inmates, besides taking a deep interest in the well-being of the institution; and they are always ready to consider any request or suggestion that may be made to them.

III.—*That all Nurses should be Hospital-trained.*

It is much to be regretted that their Lordships did not personally inspect the establishment at Putney; had they done so, they would have seen the class of patients whose needs the management are called on to supply and the class of nurses engaged. What is required is a kind personal attendant under the supervision of a skilled nurse, and this is what is given. It is difficult to obtain for the incurables even such trained nurses as are required, for the obvious reason that the scope is too limited for the exercise of their skill. There is a staff of hospital-trained nurses, and under them are the assistant nurses, concerned with the daily care and the daily wants of their patients, and each under the oversight and direction of the hospital-trained or "divisional" nurse. The menial work is done by ward-maids. Many of the ordinary nurses have been several years in the institution; they are perfectly trained for its peculiar work, and are to be commended for efficiency and kindness to their patients. For such reasons the Board think that these nurses have an advantage over professional nurses.

IV.—*That the contracts for food and stores of all kinds should be by open tender.*

The question of throwing open, by advertisement, the contracts for provisions admits of different opinions. It secures cheapness in price; but, where quality is the object, the result is doubtful. For many years bread was supplied by contract; the experience was so unsatisfactory, that provision was at length made for making bread on the premises. Milk is almost entirely the produce of cows kept at the institution. For meat, butter, cheese, bacon, groceries, coals, soap, &c., a limited number of tradesmen, all of good repute, are invited every quarter to tender. It is perhaps not the cheaper method, but it goes far to secure articles of uniform good quality with due regard to economy.

V.—*That the general supervision by the Committee of Governors should be greatly increased.*

The Board of Management meet fortnightly. The House Committee attend at the hospital every Wednesday for several hours; they see the medical officer, the Matron, and the steward, and receive reports from each and see their journals; they consider requests and complaints; they inspect the building, and visit the patients at their meals. Members of the committee, either personally or by their families, attend at frequent intervals during the week, and are in direct communication with the inmates.

* It appears that in a general metropolitan hospital the number of cases requiring specific medical treatment is 90 per cent.

VI.—*The authorities of the hospital appear to be incapable of effecting reforms, and are extremely resentful of external observation.*

If it is necessary to meet general remarks of this kind, the Board would take leave to point to the institution itself, which, by consent of persons really acquainted with it and who have visited it, is allowed to be equal if not superior in its management, order, and appointments to any other charitable institution. It has been visited by delegates from Scotland, Ireland, Manchester, the Dominion of Canada, the Australian Colonies, and foreign countries, who have all expressed not only their unqualified approval, but their admiration of its administration. It is not conducted upon the lines of a general hospital nor of a workhouse infirmary, which were continually present to theminds of their Lordships; it is neither the one nor the other, and should be judged, not by comparison with them, but on its own merits. The Board, some of whom have watched its development from its cradle at Carshalton to the present time, appeal from a tribunal the members of which have never visited the institution, to their subscribers.

*Conclusion.*

From the above it will be seen that the Board do not admit the justice or validity of their Lordships' conclusions, and they submit that their subscribers and the public will be of the same opinion as themselves.

It is to be regretted that the Committee, before entering on the inquiry, had not invited information from those most competent to give it, namely, from the Board and their officers. This information would have been cheerfully given, and would have obviated many of their Lordships' objections and prevented much of the misunderstanding which evidently existed in their Lordships' minds with reference to the management of this institution.

